# Ethnic and Sex Differences on Neuropsychological Measure Predictions of Functional Decline

**DOI:** 10.1002/alz70857_103886

**Published:** 2025-12-25

**Authors:** Genna M Mashinchi, Taylor F Levine, Emily Post, Jessica ZK Caldwell

**Affiliations:** ^1^ Cleveland Clinic, Las Vegas, NV, USA; ^2^ Cleveland Clinic Lou Ruvo Center for Brain Health, Las Vegas, NV, USA

## Abstract

**Background:**

Alzheimer's disease and related dementias (ADRD) are measured on a continuum. Neuropsychological evaluations, which assess cognitive abilities that predict functional independence, are bottlenecked by limited resources. Identifying tests sensitive to early functional decline can streamline evaluations. Women and Hispanic Latinos (HL) have higher ADRD risk compared to men and non‐Hispanic whites (NHW). We examined ethnic and sex differences in the relationship between neuropsychological measures and functional decline in a NHW and HL sample.

**Method:**

Data included 661 self‐reported NHW and 631 HL participants from Health and Aging Brain Study‐Health Disparities who completed baseline and follow‐up testing (18‐24 months post‐baseline) and were cognitively normal (Clinical Dementia Rating Scale [CDR]=0) or mildly cognitive impaired (CDR=0.5) at baseline. Building on Jutten (2021), hierarchical linear models examined the relationship between baseline neuropsychological performance (Trail Making Test B [TMTB], animal fluency, Spanish English Verbal Learning Test delayed recall) and functional decline (CDR‐Sum of Boxes [CDR‐SB]), hypothesizing that TMTB would best predict functional decline for both cohorts.

**Result:**

In NHW, all measures predicted functional decline (*p*s < .001). Sex moderated the relationship between TMTB and functional change (*p* = .043). When stratified by sex, both NHW men and women with worse TMTB performance demonstrated greater functional decline (*p*s < .001). In men, worse TMTB related to worse functioning for those with average or below‐average TMTB performance. This was similar for women with average or below‐average TMTB; however, women with the fastest TMTB showed the opposite relationship. In the HL cohort, only TMTB predicted functional decline (*p* < .001), and sex significantly moderated this relationship (*p* = .022). HL men showed greater functional decline with worse TMTB (*p* = .002), whereas HL women showed preserved functional ability regardless of TMTB (*p* = .082).

**Conclusion:**

These findings show good prediction of functional decline with worse neuropsychological performance on several measures in NHW, but only with TMTB in HL. In both groups, the TMTB/functional decline relationship was stronger in men than women. These findings illustrate the sensitivity of TMTB to functional decline across NWH and HL groups, highlighting clinical implications for referral screeners. Furthermore, greater functional decline prediction in men supports literature suggesting need for more sensitive assessment in women.

